# Chemists' Scholarships Go Begging

**Published:** 1920-08-28

**Authors:** 


### Chemists' Scholarships Go Begging.

Regret is expressed by the Pharmaceutical Council of Great Britain that only two candidates entered this year for the Jacob Bell scholarships, which are awarded annually to registered students of the Pharmaceutical Society.

Neither of the candidates obtained a sufficient number of marks to reach the standard, and the scholarship has therefore not been awarded this year.

These scholarships were founded in memory of Jacob Bell, one of the original founders of the Pharmaceutical Society, and entitle the holders to free tuition at the Bloomsbury Square College of Pharmacy, together with a gift of books. While the result this year is disappointing, it is no doubt due to temporary causes, and it is hoped that once more enthusiam will be shown in regard to these scholarships.

